# Estimating the nursing costs for medical in-patients using the MY-DRG at a teaching hospital: the Universiti Kebangsaan Malaysia experience

**DOI:** 10.1186/1471-2458-14-S1-O14

**Published:** 2014-01-29

**Authors:** Nor Haty Hassan

**Affiliations:** 1 United Nations University International Institute for Global Health (UNU-IIGH), 56000 Cheras, Kuala Lumpur, Malaysia

## Background

Nursing care is a major portion of routine hospital care, especially when compared to more ancillary services such as labs, drugs, radiology, and other diagnostic and therapeutic procedures performed by doctors at the hospital. Although the nursing profession had been developed in Malaysia since the 1900s, the current hospital billing system does not reflect what nurses provide for their patients. To date, nursing service costs are poorly estimated in Malaysian economic studies. Nursing services are commonly lumped together into the patient’s total charge per day in the hospital, and billed as a flat daily rate, based on the type of room they occupy. The primary objective of this study was to calculate the inpatient nursing costs in six medical wards, which provide varied specialty services for UKMMC’s inpatients.

## Materials and methods

A descriptive cross-sectional design was applied and a top down costing form was used to calculate the nursing costs (unit/day). The data comprise of: total number of staff nurses and their grades; salary received per year (including allowances); patient days per year; and average length of hospital stay (ALOS) in 2011. These data were analysed using SPSS version 21.

## Results

The nursing unit costs per day for inpatients were identified: medical (RM101); surgical (RM151); paediatric (RM199); obstetrics and gynaecology (RM219); orthopaedic (RM115); psychiatric (RM151); ENT (RM130) and ophthalmology (RM164). The top five highest mean costs for nursing components with MY-DRG for medical inpatients were: A-4-10-III (septicaemia severe) (RM1300.98, SD±811); A-4-14-III (other bacterial and parasitic infections severe) (RM1264.9, SD±598.9); A-4-10-II (moderate septicaemia) (RM927, SD±384.8); A-4-14-II (other bacterial and parasitic infections moderate (RM721, SD±345) and finally A-4-13-III (viral and other non-bacterial infections severe) (RM663, SD±266.5).

## Conclusions

The present study only took nurses’ salaries into account. However, further justifications are needed to calculate the nursing costs using top down costing, in order to achieve a more accurate and cohesive result.

